# Long-term survival in patients with metastatic head and neck squamous cell carcinoma treated with metastasis-directed therapy

**DOI:** 10.1038/s41416-019-0601-8

**Published:** 2019-10-25

**Authors:** Thomas H. Beckham, Jonathan E. Leeman, Peng Xie, Xiaolin Li, Debra A. Goldman, Zhigang Zhang, Eric Sherman, Sean McBride, Nadeem Riaz, Nancy Lee, C. Jillian Tsai

**Affiliations:** 1 Department of Radiation Oncology, Memorial Sloan Kettering Cancer Center, New York, NY USA; 2 Department of Epidemiology and Biostatistics, Memorial Sloan Kettering Cancer Center, New York, NY USA; 3 Department of Medicine, Memorial Sloan Kettering Cancer Center, New York, NY USA

**Keywords:** Metastasis, Head and neck cancer

## Abstract

**Background:**

Our objective was to evaluate the outcomes of metastatic head and neck squamous cell carcinoma (HNSCC) by disease burden with an emphasis on metastasis-directed therapy (MDT) in patients with limited metastatic disease burden.

**Methods:**

In total, 186 patients who developed metastatic disease after definitive therapy for HNSCC were included. Clinically and radiographically apparent metastases were enumerated. Kaplan–Meier methods were used to estimate survival. Cox regression was used to assess the association between clinical variables.

**Results:**

Patients with a single metastasis had a 5-year overall survival (OS) of 35% (95% CI 16–54%) in contrast to patients with multiple metastases with a 5-year OS of 4% (95% CI 2–9%). Thirty patients (16.1%) underwent MDT. On multivariable analysis, oral cavity or sinonasal primary (HR 2.22 95% CI 1.16–4.25, *p* = 0.015; HR 4.88, 95% CI 1.10–21.70, *p* = 0.037, respectively) were associated with higher risk of death, whereas receipt of MDT (HR 0.36, 95% CI 0.17–0.74, *p* = 0.006) was associated with lower hazard of death. Median subsequent metastasis-free survival and 5-year survival after MDT (*n* = 30) were estimated at 26.4 months (95% CI: 9.8–54.0) and 31%, (95% CI: 15–48%).

**Conclusions:**

HNSCC patients with limited metastatic disease may derive significant benefit from MDT. Prospective trials evaluating MDT in HNSCC are warranted.

## Background

Distant metastatic disease occurs in ~15% of HNSCC patients after initial definitive management.^1–3^ Patients can present in various states from a single site of metastasis and controlled local disease to widely disseminated metastases with or without local recurrence. Despite the intuition that these patients may have very different clinical trajectories, there is little guidance on whether management of HNSCC patients should be tailored based on metastatic disease burden.

Mounting scientific and clinical evidence has accumulated since initial proposal of the oligometastatic state by Hellman and Weichselbaum in 1995.^4^ There has been a dramatic increase in the understanding of the highly complex process of metastasis initiation and metastatic outgrowth.^5^ The increased understanding of the complexity and variability of metastatic biology has been accompanied by increasing reports of success using MDT to treat patients with oligometastatic disease.

Recently, prospective studies examining the role of local therapy in managing metastatic disease for modification of disease outcome has clarified a long-running debate about the role of local therapy in patients with limited metastatic disease burden.^6^ Randomised phase II trials have demonstrated meaningful benefits of metastasis-directed therapy (MDT): androgen deprivation therapy-free survival in oligometastatic prostate cancer,^7^ overall survival in patients treated with consolidative radiotherapy after chemotherapy for oligometastatic non-small cell lung cancer^8^ and overall survival in multiple histologies treated with ablative radiation therapy versus physician choice systemic therapy.^9^ However, as none of these studies focused on patients with HNSCC, we reviewed the outcomes of patients treated at our centre for locally advanced HNSCC who subsequently developed metastatic disease and analysed the outcomes of patients with limited metastatic disease who underwent MDT.

## Methods

### Patients

Patients treated at Memorial Sloan Kettering Cancer Center with either definitive or adjuvant local radiotherapy for HNSCC including oropharynx (OPC), oral cavity (OC), larynx, hypopharynx, nasopharynx (NPC), sinonasal and unknown primary between 1989 and 2014 were eligible for inclusion under an Institutional Review Board approved retrospective protocol. All patients who developed metastatic disease after the completion of their initial definitive treatment were included in this analysis. Patients with metastatic disease at the time of definitive local therapy for their HNSCC were excluded. Patients were considered metastatic based on pathology (biopsy or resection) or based on radiology alone in cases where biopsy was not performed, and imaging strongly suggested metastatic disease. In cases of lung metastases, available histopathological information such as concordance of HPV and or p16 status with primary disease, absence of in situ component and pathologist’s impression were used to determine whether the lung disease more likely represented metastasis versus a new primary lung tumour. Non-concordant HPV/p16 status, in situ disease and pathologist conclusion that disease was more likely new primary than metastatic disease were not included. The number of sites and total number of clinically and radiographically apparent metastatic disease at distant metastatic diagnosis were enumerated.

### Follow-up after initial local therapy

After completion of radiotherapy for local disease, patients were evaluated every 2 to 3 months for the first 2 years following treatment, and subsequently every 4 to 6 months. Follow-up visits consisted of a physical exam and flexible fibreoptic endoscopy. Three months after treatment, PET/CT, CT or MRI of the neck were performed. Afterwards, imaging studies were performed as clinically indicated.

### Statistical considerations

Overall survival (OS) was calculated from time of distant metastases (DM) until death. Patients alive at last follow-up were censored. In patients who received MDT, subsequent metastases-free survival (SMFS) was calculated from time of distant metastases (DM) until development of new lesions or death. Patients alive without additional metastases by last follow-up were censored. Kaplan–Meier methods were used to estimate median and annual outcomes with 95% confidence intervals (CI). The number of metastases was analysed two ways: (1) 1, 2, 3, 4, 5 + and (2) single vs. multiple. Based on the Kaplan–Meier findings, the number of metastases categorised as single vs. multiple was included in multivariable analyses.

Univariable and multivariable Cox regression were used to assess the relationship between number of metastases and other potential confounding factors with OS. Additional factors examined included gender, number of organs with metastasis (single vs. multiple), metastasis location (lung only, lymph node only, all others), age at DM, Karnofsky performance status (KPS)^10^ at DM, months between initial diagnosis and DM (logarithmically transformed), original tumour location (oropharynx (OPC), oral cavity (OC), nasopharynx (NPC), sinonasal, all others), metastases location (lymph nodes only, lung only, all others), local control at the time of DM (yes vs. no), Charlson comorbidity index (CCI), chemotherapy after DM (yes vs. no) as a time-dependent factor and MDT (yes vs. no) as a time-dependent factor. Due to the high degree of missing data in HPV/p16 status (78%), this factor could not be explored in multivariable analyses. All other variables were included in multivariable analyses regardless of significance. However, as number of organs and metastasis location were essentially measuring the same factor (single versus multiple sites) and were found to be highly associated, we did not include metastasis location in the multivariable model. Furthermore, because of the overlapping definitions of number of organs with metastases with number of metastases (a patient with singular metastasis could not have multiple organs with metastases), we only included number of metastases in the multivariable model.

We assessed differences in clinical characteristics between patients who did and did not receive MDT with Fisher’s Exact test and the Wilcoxon rank- sum test. In OPC patients, we analysed the relationship between HPV/P16 status and OS with Kaplan–Meier (KM) methods, both as a complete case analysis, and including missing as a covariate level.

Two-sided *p*-values < 0.05 were considered statistically significant. All analyses were performed with SAS 9.4 (The SAS Institute, Cary, NC).

## Results

### Patient and clinical characteristics

In total, 186 patients were included. Median patient age at DM was 61.7 years (range: 28.5–91.9) and 37 patients (20%) were female. Of the 186, 178 had KPS available at DM with a median of 80 (range, 40–100). In OPC patients, 42 (48%) were HPV or p16-positive, 15 (17%) were negative and 31 (35%) had unknown status. Please see Table 1 for more information.

**Table 1:** Patient characteristics

		*N* (%)
# Patients		186
Age at DM, years	Median (range) (*n* = 186)	61.7 (28.5–91.9)
Sex	Male	149 (80.1)
	Female	37 (19.9)
Race	White	135 (72.6)
	Asian	22 (11.8)
	Black	14 (7.5)
	Other	2 (1.1)
	Unknown	13 (7)
BMI	Median (range) (*n* = 171)	26.7 (13.4–41.1)
Histology	SCC	186 (100)
T Stage	T1	21 (11.3)
	T2	65 (34.9)
	T3	40 (21.5)
	T4	51 (27.4)
	Unknown	9 (4.8)
N stage	N0	27 (14.5)
	N1	24 (12.9)
	N2	130 (69.9)
	N3	5 (2.7)
Initial AJCC stage	I	1 (0.5)
	II	12 (6.5)
	III	30 (16.1)
	IV	143 (76.9)
KPS at diagnosis	Median (range) (*n* = 178)	90 (60–100)
KPS at distant metastases	Median (range) (*n* = 178)	80 (40–100)
CCI at DM	Median (range) (*n* = 186)	8 (6–11)
HPV/P16 status in OPC	Positive	42 (47.7)
	Negative	15 (17)
	Unknown	31 (35.2)

*DM* distant metastasis, *BMI* body mass index, *KPS* Karnosfky performance status, *CCI* Charlson comorbidity index, *HPV* human papillomavirus, *OPC* oropharyngeal carcinoma

Metastasis details are listed in Table 2. The median time between initial cancer diagnosis and DM was 13.1 months (range: 2.7–80.8). This variable was sufficiently skewed, so time was logarithmically transformed for formal analyses. Twenty-five patients (13%) had a solitary metastasis, 34 patients (18%) had two metastases, 25 patients (13%) had 3 metastases, 20 patients (11%) had 4 metastases and 82 patients (44%) had 5 or more metastases. The most common site of metastases was the lung (138/186, 74%), followed by non-cervical lymph nodes (75/186, 40%).

**Table 2:** Distant metastasis details and subsequent treatment characteristics

		*N* (%)
Months between original diagnosis and metastasis	Median (range) (*n* = 186)	13.1 (2.7–80.8)
# of Metastases	1	25 (13.4)
	2	34 (18.3)
	3	25 (13.4)
	4	20 (10.8)
	5+	82 (44.1)
# Organs with metastases	1	99 (53.2)
	2	66 (35.5)
	3	13 (7)
	4	8 (4.3)
*Location of metastases*		
Adrenal		1 (0.5)
Bone		40 (21.5)
Bowel		2 (1.1)
Brain		3 (1.6)
Heart		2 (1.1)
Kidney		2 (1.1)
Liver		23 (12.4)
Lung		138 (74.2)
Non-cervical node		75 (40.3)
Skin		6 (3.2)
Soft tissue		10 (5.4)
Metastasis-directed therapy	Surgery	26 (14)
	RT	3 (1.6)
	RFA	1 (0.5)
	None	156 (83.9)
Months between DM and metastasis-directed therapy	Median (range) (*n* = 30)	0.9 (0.0–16.1)
Chemotherapy for metastatic disease	None	54 (29)
	Cetuximab	54 (29)
	Other chemo	78 (41.9)
Months between DM and chemotherapy	Median (range) (*n* = 132)	1.2 (0.0–65.4)

*DM* distant metastasis, *RT* radiation therapy, *RFA* radiofrequency ablation

### Overall survival estimates

**Fig. 1:**
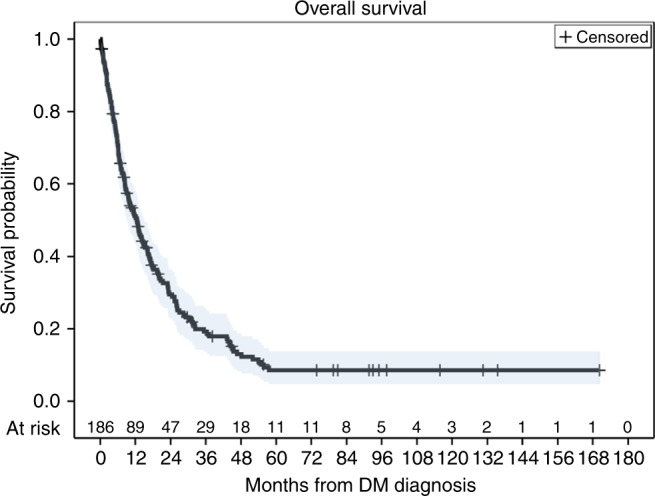
Kaplan–Meier plot for overall survival for all patients after diagnosis of distant metastatic disease. Blue shading indicates 95% confidence interval of the estimate

By the end of follow-up 156 patients had died with a median OS estimate of 12.5 months (95% CI: 9.0–15.2 months) after diagnosis of DM. One-year and 5-year estimates were 51% (95% CI: 44–58%) and 9% (95% CI: 5–14%), respectively (Fig. 1). Median follow-up in survivors was 30.5 months (range: 0.5–170.2 months).

### The relationship between clinical factors and metastasis treatment and OS

**Fig. 2:**
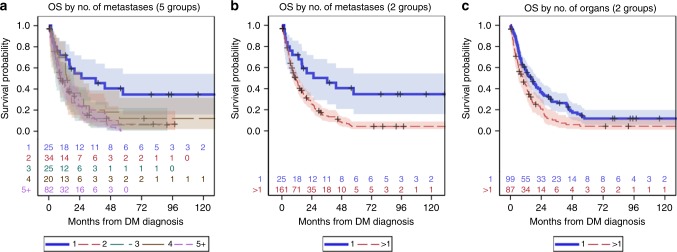
Kaplan–Meier plots for overall survival (OS) according to (**a**) the number of metastases separated into five groups, (**b**) the number of metastases separated into two groups (single versus multiple), (**c**) the number of organs involved with metastases. Shading indicates the 95% confidence interval for the estimate

**Fig. 3:**
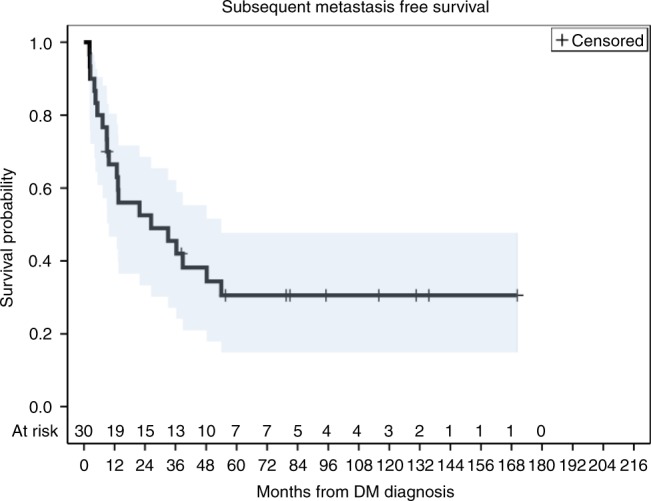
Kaplan–Meier plot for subsequent metastasis-free survival measured from the time of metastasis-directed therapy. Shading indicates 95% confidence interval

**Fig. 4:**
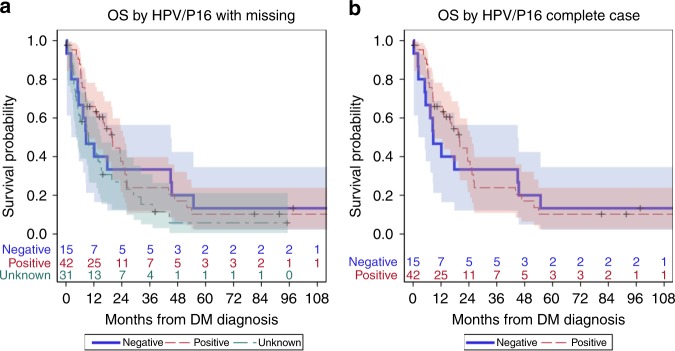
Kaplan–Meier plots for overall survival in patients with oropharyngeal cancer separated by HPV/p16 status including (**a**) patients with unknown status considered as a separate group and (**b**) patients with unknown status excluded. Shading indicates 95% confidence interval

As Fig. 2 illustrates, the survival distributions of 2, 3, 4 and 5 + (Fig. 2a) and 2–4 and 5 + (Fig. 2b) overlap, suggesting no significant difference in survival distribution for these groupings. When comparing single versus multiple metastases, the 5-year OS of patients with single metastases was 35% (95% CI: 16–54%) compared with 4% (95% CI: 2–9%) for patients with multiple metastases. Patients with multiple metastases had a higher risk of death (HR: 2.58, 95% CI: 1.50–4.43, *p* < .001) compared with patients with a single metastasis.

Univariable and multivariable analyses of clinicopathologic features and OS are presented in Table 3. In univariable analysis, having multiple organs with metastases (HR: 1.67, 95% CI: 1.21–2.29, *p* = 0.002) was associated with a higher risk of death. Patients with OC (HR: 4.03, 95% CI: 2.37–6.86, *p* < 0.001) had a higher risk of death compared to patients with OPC. Higher KPS at DM was associated with a lower risk of death (HR: 0.93, 95% CI: 0.92–0.95, *p* < .001) and longer times between diagnosis and metastases were associated with a decreased risk of death (HR: 0.64, 95% CI: 0.50–0.82, *p* < 0.001). Patients who received MDT were at a lower risk of death (HR: 0.26, 95% CI: 0.15–0.46, *p* < 0.001).

**Table 3:** Cox regression analysis for relationship between patient and treatment characteristics and overall survival

		Univariable				Multivariable *n* = 178		
		*N* (#D)	HR	(95% CI)	*p*-value	HR	(95% CI)	*p*-value
# Metastases	Multiple	161 (141)	2.58	(1.5–4.43)	**<**.001	1.68	(0.84–3.40)	0.14
	Single	25 (15)	REF			REF		
# Organs with Mets		186 (156)	1.43	(1.19–1.72)	**<**0.001	---		
# Organs w Mets (2 groups)^	Multiple	87 (75)	1.67	(1.21–2.29)	0.002	---		
	Single	99 (81)	REF			---		
Definitive treatment*	Yes		0.26	(0.15–0.46)	**<**0.001	0.36	(0.17–0.74)	0.006
	No		REF			REF		
Age at DM, years		186 (156)	1.01	(0.99–1.02)	0.20	1.01	(0.98–1.03)	0.70
Sex	Female	37 (31)	1.24	(0.83–1.83)	0.29	1.14	(0.73–1.77)	0.57
	Male	149 (125)	REF			REF		
KPS at distant metastases		178 (149)	0.93	(0.92–0.95)	**<**0.001	0.95	(0.93–0.96)	**<**0.001
HN cancer site	OC	20 (19)	4.03	(2.37–6.86)	**<**0.001	2.22	(1.16–4.25)	0.015
	Sinonasal	5 (5)	1.40	(0.56–3.47)	0.47	4.88	(1.10–21.70)	0.037
	All_Others	46 (37)	0.98	(0.66–1.45)	0.91	0.94	(0.61–1.46)	0.79
	NPV	27 (24)	0.93	(0.59–1.48)	0.77	0.98	(0.59–1.64)	0.94
	OPC	88 (71)	REF			REF		
Metastases location^	All_Others	107 (92)	REF			---		
	Lung_Only	67 (56)	0.63	(0.45–0.88)	0.007	---		
	LN_Only	12 (8)	0.59	(0.29–1.22)	0.16	---		
Log time bw diagnosis & Mets		186 (156)	0.64	(0.5–0.82)	**<**0.001	0.68	(0.52–0.89)	0.005
CCI at DM		186 (156)	1.11	(0.99–1.26)	0.08	0.96	(0.77–1.19)	0.68
Local control at DM	Yes	141 (115)	0.44	(0.31–0.64)	**<**0.001	0.82	(0.54–1.25)	0.36
	No	45 (41)	REF			REF		
Chemotherapy*	Yes		2.05	(1.36–3.1)	**<**0.001	0.88	(0.56–1.40)	0.60
	No		REF			REF		
Chemotherapy type*	Cetuximab		1.49		0.07	---		
	None		REF			---		
	Other chemo		2.06		0.001	---		

*N* total sample size, *D*   number of events, *HR* hazard ratio, *CI* confidence interval, *KPS* Karnofsky performance status, analysed continuously, *HN* head and neck, *OC* oral cavity, *NPV* nasopharynx, all other HN Cancer Sites: hypopharynx, larynx, unknown primary; all other locations: all other possible combinations of metastases, *CCI* Charlson comorbidity index, *REF* reference level

*n*  =  178 due to missing data

^Not included in multivariable model due to collinearity/overlapping definitions

*Treated as an time-dependent covariate *N* not included for these covariates as the *N* vary with time

The number of metastases, HN cancer site, histology, KPS, time between initial and DM diagnosis, systemic treatment, MDT, age, gender, CCI and local control were included in the multivariable model for 178 patients with complete data. The number of organs with metastases and metastases location were essentially measuring the same concept, so metastases location was not included in the multivariable model. In addition, due to the overlapping definitions of number of metastases with number of organs with metastases, only number of metastases was included in the multivariable model. After controlling for these covariates, the number of metastases was no longer significantly associated with OS (HR: 1.68, 95% CI: 0.84–3.40, *p* = 0.14). KPS, HN cancer site, time between initial and DM diagnosis and MDT remained significant predictors of OS as well; however, local control and chemotherapy receipt after DM were not significantly associated with OS in multivariable analyses (Table 3).

### Factors associated with MDT

We examined relevant clinical features of patients who did and did not receive MDT in Table 4. Patients with MDT had a higher KPS (median: 90, range: 70–100) compared with patients who did not have MDT (median: 80, range: 40–100, *p* = 0.001). In addition, a lower proportion of patients who received MDT had >1 metastases (40%, 12/30) compared with patients who did not receive MDT (96%, 149/156), *p* < 0.001. Seven MDT patients had two metastases, two patients had four metastases and three patients had five or more metastases. Only 17% of patients (5/30) who received MDT had more than one organ involved with metastases compared with 53% of patients (82/156) who did not receive MDT (*p* < 0.001).

**Table 4:** Clinical characteristics stratified by definitive treatment status

		Definitive treatment			
		All	No	Yes	
		*N* (%)	*N* (%)	*N* (%)	*p*-value
Age at DM, years	Median (range)	61.7 (28.5–91.39)	61.9 (28.5–91.9)	60.3 (38.2–82.9)	0.48
KPS at distant metastases	Median (range)	80 (40–100)	80 (40–100)	90 (70–100)	0.001
BMI	Median (range)	26.7 (13.4–41.1)	27.0 (13.4–41.1)	25.8 (18.2–34.2)	0.21
Sex	Male	149 (80.1)	126 (80.8)	23 (76.7)	0.62
	Female	37 (19.9)	30 (19.2)	7 (23.3)	
CCI at DM	Median (range)	8 (6–11)	8 (6–11)	8 (6–11)	0.58
# Metastases (2 groups)	Single	25 (13.4)	7 (4.5)	18 (60)	**<**0.001
	Multiple	161 (86.6)	149 (95.5)	12 (40)	
# Organs w Mets (2 groups)	1	99 (53.2)	74 (47.4)	25 (83.3)	**<**0.001
	>1	87 (46.8)	82 (52.6)	5 (16.7)	
Chemotherapy	No	46 (24.7)	33 (21.2)	13 (43.3)	0.022
	Yes	132 (71)	115 (73.7)	17 (56.7)	
	Unknown	8 (4.3)	8 (5.1)	0 (0)	

*N* number, *CCI* Charlson comorbidity index, *DM* distant metastasis

Unknown value listed in table for description, but not included in the formal statistical comparison

### Subsequent metastasis-free survival

Thirty patients received MDT. Of these, 20 had a SMFS event with a median SMFS of 26.4 months (95% CI: 9.8–54.0 months). Five-year SMFS was 31% (95% CI: 15–48%) (Fig. 3). The median SMFS for patients with a single metastasis was 13.4 months (95% CI: 5.3–38.8). The median SMFS for patients with 2–4 metastases was 11.2 months (95% CI: 7.9–13.8) and median SMFS for patients with five or more metastases was 8.2 months (95% CI: 5.9–11.5).

### HPV/p16 status in OPC patients

No significant association was found between HPV/P16 status and OS, either as a complete case analysis (*p* = 0.74) or including unknown as a covariate level (*p* = 0.18). Patients with HPV/P16-positive had a median OS of 20 months (95% CI: 9.0–25.3 months) compared with 8.6 (95% CI: 2.3–45.0 months) in HPV-negative patients. Patients with missing HPV/P16 status had a median of 9.7 months (95% CI: 4.4–15.2 months) (Fig. 4).

## Discussion

We found that patients who were selected for MDT for metastatic disease from LA-HNSCC had a significantly higher OS than patients who did not receive MDT. Patients with a single metastasis had greater OS and were most commonly selected for MDT, but the results from our analyses suggested that even patients with multiple metastases could derive clinically meaningful benefit from MDT. Below we discuss the relevant literature and highlight a number of points are necessary to consider when interpreting the findings of this report.

Several other reports on the outcome of pulmonary metastastectomy for head and neck cancers have been published. The largest study from the Metastatic Lung Tumour Study Group of Japan registry database reported 114 patients with HSNCC who underwent pulmonary metastastectomy. They reported a 5-year OS of 26.5%, with OC primary, lymph node metastasis and male sex associated with worse survival on multivariable analysis.^11^ An earlier report from Memorial Sloan Kettering Cancer Center was the largest institution-level report, with 41 patients undergoing pulmonary resection for HNSCC.^12^ Similar to this study, HNSCC patients with MDT achieved long-term survival in ~20% of cases, and long-term survival was achievable in patients with solitary and multiple metastases. Whereas that report evaluated other cancers of the head and neck including glandular cancers, this study focuses specifically on HNSCC in a more modern era of MDT techniques with more modern systemic therapy. Another study evaluated patients who developed pulmonary metastases after primary head and neck cancer therapy with no evidence of extrapulmonary metastases was carried out and attempted to compare the outcomes of surgical resection to best alternative chemotherapy or supportive care.^13^ They reported a three-year OS of 68% in the surgical group of 24 patients compared with 15% in the non-surgical group of 45 patients. Of note they included non-SCC histologies (17%). While receipt of surgery was significant on multivariate analysis, the selection bias inherent to who undergoes MDT is challenging to completely control for with available clinical characteristics. As with our study, MDT patients were more likely to have a solitary metastasis. These and other previous studies evaluating the outcomes of oligometastasis in head and neck malignancies has recently been the subject of an excellent review by the Groupe d’Oncologie Radiothérapie Tete Et Cou (GORTEC).^14^

In our study, patients with a single metastasis had significantly better OS when compared with two or more metastases in univariable analyses, and we did not find a significant difference in outcomes between patients with 2, 3, 4 or 5 + metastases. Patients with a single metastasis have been hypothesised to be the most likely to exhibit favourable metastatic biology which alone may explain superior survival compared to patients with more than one metastasis.^4,15,16^ However, in our multivariable model controlling for receipt of MDT and the number of metastases, solitary versus multiple metastases was not associated with OS. This may be due to the fact that the majority of patients (18/25) with a solitary metastasis underwent MDT. However, patients with 2–4 metastases receiving MDT had a median SMFS of 11.2 months, and long-term disease-free survival was attained in some patients. Notably, MDT remained significantly associated with OS on multivariable analysis. As such, we propose that although our study lacks sufficient power to confirm statistically, well-selected patients with more than one metastasis may still benefit from MDT, and that the exceptional OS for patients with a solitary metastasis is in part related to their propensity to receive MDT. Other features associated with improved OS on multivariable analysis in our study include longer time between cancer diagnosis and DM and higher KPS at DM. Longer time to metastatic diagnosis has been reported in other studies to be associated with improved survival,^12,13,17,18^ however, we are unaware of additional studies controlling for performance status. Patients with a longer time between initial local therapy and development of metastatic disease, particularly those who develop oligometastatic disease are likely to be those with the most favourable metastatic biology and are the patients who should be most strongly considered for MDT and considered as factors when designing trials for oligometastatic disease.

The degree to which definitive local therapy of metastatic disease impacts survival is difficult to address in a retrospective population due to obvious biases in patient selection for MDT. The majority of our patients with a single metastasis had MDT, which weakens a comparison between those with a single metastasis treated definitively and those who were not. However, our observed 10-year SMFS of 31% leads to the proposition that MDT represents an opportunity for cure of metastatic disease in well-selected patients with HNSCC, a prospect not expected with systemic therapy or supportive care. The converse finding that many patients develop subsequent metastases or die rapidly, 33% at 1 year and 54% at 3 years, highlights the fact that these patients who are clinically similar and deemed appropriate for MDT may have radically different outcomes after MDT. This likely reflects differing underlying metastatic biology and means to differentiate between these clinically oligometastatic patients with vastly different post-MDT outcomes are needed as we seek to personalise cancer care.

The majority of our patients were treated with surgery, which is an excellent modality for managing oligometastatic disease in certain locations such as the lung and has the advantage of permitting tissue diagnosis at the time of resection. The improvement in image-guided radiotherapy and increasing clinician comfort with ablative radiation therapy in managing both primary disease in the lung^19^ and metastases^20,21^ offers another approach to managing oligometastatic disease which is highly effective, non-invasive, well tolerated and not limited by sites which are less easily resected yet frequently sites of metastasis such as bone. Indeed, the highest level evidence available for MDT to date come from the SABR-COMET^9^ and Gomez^8^ Phase II trials. Importantly, these studies have both recently reported an OS benefit to MDT with ablative radiation.

The limitations of this study warrant serious consideration in the interpretation of these findings. There is great variability in our patient sample in terms of their initial disease status, disease status at metastasis, competing comorbidities and physician and personal preferences, all of which influence the selection of therapies in the setting of metastasis. We have attempted to control for these factors as rigorously as possible including validated indices of comorbidity such as CCI and KPS, but we do not propose that all bias is eliminated. Many patients received systemic therapies, and the study period runs from prior to cetuximab, which has been shown to have a survival benefit in metastatic HNSCC,^22^ into the advent of immunotherapy, which has subsequently become a component of the standard of care for platinum-refractory disease.^23^ The vast heterogeneity in systemic therapies, duration of treatment, and number of therapies prevents rigorous incorporation of the impact of these treatments into the current study.

## Conclusions

The outcome of metastatic disease in HSNCC appears to be highly variable. The observation that some patients with apparently oligometastatic disease receiving MDT undergo rapid disease progression while others are cured highlights a spectrum of metastatic biology that has yet to be meaningfully defined. MDT offers an opportunity for long-term disease-free survival for select HNSCC patients and should be considered in treating patients with limited metastatic disease. Randomised trials evaluating the value of MDT in HSNCC are warranted.

## Data Availability

Data are relevant to MSKCC patients and not publicly available. We will make de-identified data available upon request after institutional approval.

## References

[1] Leeman JE, Li J-G, Pei X, Venigalla P, Zumsteg ZS, Katsoulakis E (2017). Patterns of treatment failure and postrecurrence outcomes among patients with locally advanced head and neck squamous cell carcinoma after chemoradiotherapy using modern radiation techniques. JAMA Oncol..

[2] Tiwana MS, Wu J, Hay J, Wong F, Cheung W, Olson RA (2014). 25 year survival outcomes for squamous cell carcinomas of the head and neck: population-based outcomes from a Canadian province. Oral Oncol..

[3] Pignon J-P, le Maître A, Maillard E, Bourhis J, MACH-NC Collaborative Group. (2009). Meta-analysis of chemotherapy in head and neck cancer (MACH-NC): an update on 93 randomised trials and 17,346 patients. Radiother Oncol..

[4] Hellman S, Weichselbaum RR (1995). Oligometastases. J. Clin. Oncol..

[5] Weichselbaum, R. R. The 46th David A. Karnofsky memorial award lecture: oligometastasis-from conception to treatment. *J. Clin. Oncol.* **36**, 3240–3250 (2018). 10.1200/JCO.18.00847 30260759

[6] Palma DA, Salama JK, Lo SS, Senan S, Treasure T, Govindan R (2014). The oligometastatic state - separating truth from wishful thinking. Nat. Rev. Clin. Oncol..

[7] Ost P, Reynders D, Decaestecker K, Fonteyne V, Lumen N, De Bruycker A (2018). Surveillance or metastasis-directed therapy for oligometastatic prostate cancer recurrence: a prospective, randomized, multicenter phase II trial. J. Clin. Oncol..

[8] Gomez DR, Tang C, Zhang J, Blumenschein GR, Hernandez M, Lee JJ (2019). Local consolidative therapy vs. maintenance therapy or observation for patients with oligometastatic non-small-cell lung cancer: long-term results of a multi-institutional, phase II, randomized study. J. Clin. Oncol..

[9] Palma, D. A., Olson, R., Harrow, S., Gaede, S., Louie, A. V., Haasbeek, C. et al. Stereotactic ablative radiotherapy versus standard of care palliative treatment in patients with oligometastatic cancers (SABR-COMET): a randomised, phase 2, open-label trial. *Lancet*. **393**, 2051–2058 (2019). 10.1016/S0140-6736(18)32487-5 30982687

[10] Yates JW, Chalmer B, McKegney FP (1980). Evaluation of patients with advanced cancer using the Karnofsky performance status. Cancer.

[11] Shiono S, Kawamura M, Sato T, Okumura S, Nakajima J, Yoshino I (2009). Pulmonary metastasectomy for pulmonary metastases of head and neck squamous cell carcinomas. Ann. Thorac. Surg..

[12] Liu D, Labow DM, Dang N, Martini N, Bains M, Burt M (1999). Pulmonary metastasectomy for head and neck cancers. Ann. Surg. Oncol..

[13] Miyazaki T, Hasegawa Y, Hanai N, Ozawa T, Hirakawa H, Suzuki A (2013). Survival impact of pulmonary metastasectomy for patients with head and neck cancer. Head Neck.

[14] Sun XS, Michel C, Babin E, De Raucourt D, Péchery A, Gherga E (2018). Approach to oligometastatic disease in head and neck cancer, on behalf of the GORTEC. Future Oncol..

[15] Reyes DK, Pienta KJ (2015). The biology and treatment of oligometastatic cancer. Oncotarget.

[16] Lussier YA, Xing HR, Salama JK, Khodarev NN, Huang Y, Zhang Q (2011). MicroRNA expression characterizes oligometastasis(es). PLoS One.

[17] Chen F, Sonobe M, Sato K, Fujinaga T, Shoji T, Sakai H (2008). Pulmonary resection for metastatic head and neck cancer. World J. Surg..

[18] Nakajima Y, Iijima Y, Kinoshita H, Akiyama H, Beppu T, Uramoto H (2017). Surgical treatment for pulmonary metastasis of head and neck cancer: study of 58 cases. Ann. Thorac. Cardiovasc. Surg..

[19] Timmerman R, Paulus R, Galvin J, Michalski J, Straube W, Bradley J (2010). Stereotactic body radiation therapy for inoperable early stage lung cancer. JAMA..

[20] Kinchen CL, Taylor TN, Johnstone CA, Robbins JR (2017). Stereotactic body radiation therapy for palliative treatment of bone metastases: practice patterns and survival outcomes. J. Clin. Oncol..

[21] Murphy JD, Nelson LM, Chang DT, Mell LK, Le Q-T (2013). Patterns of care in palliative radiotherapy: a population-based study. J. Oncol. Pract..

[22] Vermorken JB, Mesia R, Rivera F, Remenar E, Kawecki A, Rottey S (2008). Platinum-based chemotherapy plus cetuximab in head and neck cancer. N. Engl. J. Med..

[23] Ferris RL, Blumenschein G, Fayette J, Guigay J, Colevas AD, Licitra L (2016). Nivolumab for recurrent squamous-cell carcinoma of the head and neck. N. Engl. J. Med..

[24] Leeman JE, Patel SH, Anderson ES, Tsai CJ, McBride SM, Dunn L (2017). Long-term survival in oligometastatic head and neck cancer patients. J. Clin. Oncol..

[25] Leeman JE, Beckham T, Xie P, Li X, Spielsinger D, Goldman DA (2018). Metastasis Directed Therapy with Definitive Intent is Associated with Improved Survival in Metastatic Head and Neck Cancer. Int. J. Rad. Oncol.*Biol.*Phy..

